# Mask Mirroring: A Novel Approach to Healthcare Empathy in the COVID-19 Era

**DOI:** 10.7759/cureus.42185

**Published:** 2023-07-20

**Authors:** Gupta Sahil, Mark Friedrich Hurdle, Jessie N Fox, Lohit Garg

**Affiliations:** 1 Department of Pain Medicine, Mayo Clinic, Jacksonville, USA; 2 Department of Nursing Education, Mayo Clinic, Jacksonville, USA; 3 Cardiology, University of Colorado Anschutz Medical Campus, Aurora, USA

**Keywords:** covid-19, face mask wearing, patient comfort, team work and public health, masking

## Abstract

The COVID-19 pandemic has brought forth substantial changes to societal norms and global health infrastructure, one of the most impactful being mask wearing. With varying attitudes toward mask usage in a post-pandemic environment, this paper introduces the concept of "mask mirroring" in healthcare settings. This strategy involves healthcare providers reciprocating the mask-wearing behavior of their patients, intending to respect patients' choices and alleviate their potential concerns and anxieties. It is hypothesized that mask mirroring could serve as a symbol of empathy and solidarity, enhancing the doctor-patient rapport and facilitating effective healthcare delivery. In addition, it could reduce the transmission of respiratory infections, fostering a safer healthcare environment. Importantly, mask mirroring addresses the power dynamics between healthcare providers and patients, allowing patients' preferences and comfort to be prioritized. The implementation of this concept requires clear communication about its purpose and symbolism, striking a balance between reassurance and respect for differing viewpoints. The ultimate aim of mask mirroring is to promote patient-centric care, reflecting our commitment to understanding and empathizing with patients' concerns in a world recovering from COVID-19.

## Editorial

As we approach the denouement of the COVID-19 pandemic, which has reshaped our global health structure and societal norms, we must address the persisting issue of masking - now a divisive topic among patients [1]. With most of our patients now comfortable without masks, healthcare providers (HCPs) still encounter a fraction who continue to prefer this safeguard. This group’s apprehensions are certainly not unfounded. As HCPs, it's our responsibility to respect their anxieties and honor their choices.

**Figure 1:**
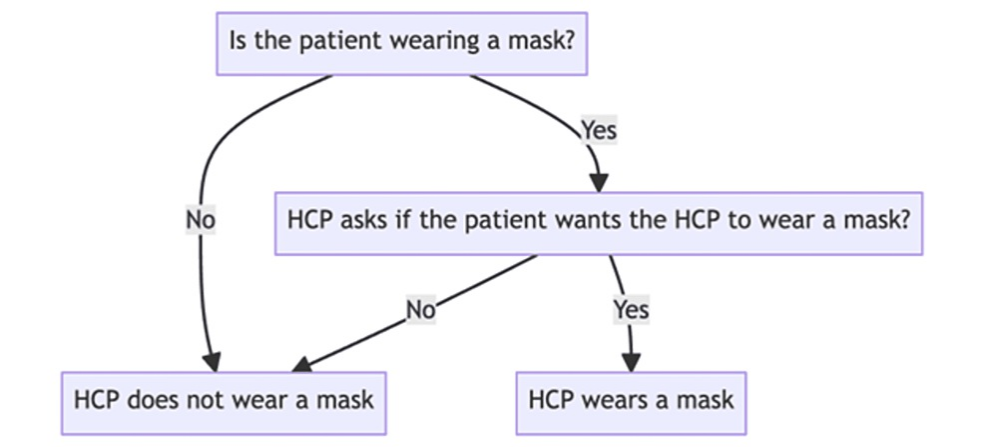
Flowchart illustrating the decision-making process for the HCP on whether to wear a mask, based on the patient’s mask wearing status and preference. HCP: healthcare provider

This leads us to a new proposal - a concept we like to call "mask mirroring” (Figure 1). This entails that if a patient enters our facilities masked, HCPs shall reciprocate by donning a mask themselves. The implementation of mask mirroring requires tact, sensitivity, and clear communication about its purpose, symbolism, and ultimate goal: to enhance patient care by fostering trust and rapport. The benefits of this initiative are multifold.

First, mask mirroring can assuage any lingering fears among patients. Even as we see light at the end of the COVID-19 tunnel, apprehensions still abound. By matching our patients' choice to mask, we communicate a crucial message: their concerns are being heard, acknowledged, and respected. This promotes an environment of mutual respect and psychological safety, which in turn could enhance the doctor-patient rapport, further facilitating effective healthcare delivery [2].

Second, mask mirroring could act as a symbolic gesture that exemplifies empathy, which is an invaluable asset in healthcare [3]. The simple act of wearing a mask demonstrates solidarity with our patients, showing them that we understand their need for precautions despite the diminished threat.

Moreover, mask mirroring carries potential health benefits. As the pandemic has highlighted, masks are effective tools in reducing the transmission of respiratory infections [4]. This practice could help protect those who are immunocompromised or are otherwise at risk, contributing to a safer healthcare environment overall. However, it is essential to note that this should not encourage excessive or unnecessary mask use. Moreover, over-masking could potentially create a false sense of security and detract from other important hygiene measures, such as handwashing. Thus, it is crucial that mask mirroring is implemented as a part of a broader, comprehensive approach to infection prevention and control, not as a standalone measure. 

An additional factor we must consider in this discourse is the inherent power differential between HCPs and patients. The patient, often in a vulnerable state of health, could be hesitant to voice their preference due to the authority that medical professionals command [5]. They might prefer their HCP to don a mask but hold back their request, perhaps fearing that speaking up could be perceived as challenging or disrespectful. The initiative of mask mirroring negates this predicament. When HCPs proactively mask in response to their patients' actions, it alleviates the burden from patients' shoulders, fostering an environment where their comfort and preference are paramount. It reassures them that their needs are prioritized and their voice, even when silent, is heard. This practice, therefore, not only serves to protect physical health but also to bolster mental well-being by empowering patients within the healthcare setting.

It is crucial to mention that mask mirroring should not be regarded as an imposition or endorsement of fear, but rather an extension of our commitment to patient-centric care. It mirrors not just the mask but the sentiment, empathizing with those still navigating their path out of pandemic-induced anxiety.

However, implementing mask mirroring requires tact and sensitivity. It involves striking a balance between reassuring those who mask without alienating those who do not. It is here that clear communication will play a critical role. HCPs should explain the reasons behind mask mirroring, its symbolism, and its ultimate goal: to enhance patient care by fostering trust and rapport.

As we step into a post-COVID world, it is paramount that we do not disregard the emotional scars left by the pandemic. Mask mirroring is an attempt to address these lingering concerns, ensuring that we move forward together in health and harmony. By mirroring our patients' choices, we demonstrate our willingness to walk that extra mile in empathy and respect, reaffirming the values of compassionate healthcare.
